# An Alternative to Antibiotics: Selected Methods to Combat Zoonotic Foodborne Bacterial Infections

**DOI:** 10.1007/s00284-021-02665-9

**Published:** 2021-10-09

**Authors:** Ewelina Łojewska, Tomasz Sakowicz

**Affiliations:** Department of Molecular Biotechnology and Genetics, Faculty of Biology and Environmental Protection, University of Lodz, Building A, Banacha 12/13 Street, 90-237 Lodz, Poland

## Abstract

Pathogenic bacteria contaminating food or animal feed cause serious economic losses in the health sector as well as is in the agriculture and food industry. The development of bacterial resistance due to the misuse of antibiotics and chemicals, especially in the farm industry, can bring dangerous effects for the global population therefore new safe biological antimicrobial solutions are urgently needed. In this paper, we investigate biological alternatives to antibiotics against foodborne pathogens. The most promising alternatives include antimicrobial proteins, bacteriophages, probiotics, and plant-based substances. Each described group of substances is efficient against specific foodborne bacteria and has a preferred use in an explicit application. The advantages and drawbacks of each method are outlined in the final section. Biological antibacterial solutions are usually easily degradable. In contrast to antibiotics or chemical/physical methods, they are also far more specific. When introducing new antibacterial methods it is crucial to check their safety and ability to induce resistance mechanisms. Moreover, it is important to assess its activity to inhibit or kill in viable but nonculturable cells (VBNC) state and biofilm forms. VBNC bacteria are considered a threat to public health and food safety due to their possibility of remaining viable and virulent. Biological alternatives to antibiotics complete the majority of the advantages needed for a safe and efficient antimicrobial product. However, further research is necessary to fully implement those solutions to the market.

## Introduction

Bacterial foodborne diseases are a serious threat when it comes to health safety. WHO states that the global median number of bacterial diseases is 360 million per year, 60% of which are foodborne diseases. A median number of 260,000 people die each year globally due to bacterial foodborne diseases [1]. Even though bacterial foodborne diseases can be prevented, they still cause a health and economic burden for every country in the world. The economic cost of all foodborne diseases in the USA is estimated at approximately $10–83 billion annually; therefore, we can assume that at least half of this cost is caused by bacterial pathogens [2].

Foodborne bacterial pathogens are usually strictly connected to animal breeding. For example, *Salmonella* and *Campylobacter* are often transmitted from poultry; moreover, these bacteria repeatedly display antimicrobial resistance phenotypes [3, 4]. In agriculture, pathogenic foodborne bacteria can affect not only the final product but also animal health and production efficiency. For the above reasons, it seems that finding an efficient method to control foodborne bacterial pathogens at the early stages of food production (e.g., during animal breeding), could also decrease the incidence of human infections [5]. For years, antibiotics have been widely used for this purpose. However, evidence of bacteria gaining resistance to antibiotics came to light, and currently, after many years of usage, residues of antibiotic drugs are being detected in meat, milk, and egg products [6]. Many of the antibiotics that have been administrated is excreted in an active form. Furthermore, there are serious concerns about the transfer of resistance genes from farms to manure, soil, water, and finally to the environment of humans [7]. Such a scenario leads to an increase in antibiotic-resistant bacteria and then also to antibiotic-resistant pathogens transmitted to humans via the environment or food. For example *Salmonella enterica* ser. Typhi infections resistant to ciprofloxacin reached 74% in 2017 from 22% in 1990, while *Shigella* infections have increased from less than 5% in 2013 to the 24% in 2017 [8]. According to the CDC report from 2019 foodborne bacteria are on the highest rise among all resistant bacteria in the last 5 years [6]. Therefore, current law concerning agricultural production significantly limits the use of antibiotics for the breeding industry [9], especially in the EU and the USA. However, in many other countries, particularly developing ones, there is still a lack of antibiotic usage regulations which can lead to the deepening of the global problem [10, 11]. Finding an efficient alternative to antibiotics that could boost the first stages of food production is an urgent need. Importantly, such treatment must be feasible and applicable across large groups of livestock. Furthermore, it should not be toxic to humans. The objectives of the review were to identify and validate the most promising natural non-antibiotic biological methods against foodborne pathogens and to describe their efficiency based on up-to-date experimental findings. Moreover, current consumer choices are progressively more conscious and aimed at natural and ecological food production methods [12]. The Discovery of relevant methods and approaches that have been successful and collecting information about current research projects can help to establish a foundation on which future research can be based. For the study, few search engines were used: Web of Science, Scopus, and Google Scholar. The research database was built by the usage of keywords such as biological alternatives to antibiotics, non-antibiotic biological agents, antibiotic-resistant foodborne pathogens.

The literature on the subject indicates a variety of novel methods to fight against bacteria such as antimicrobial proteins (AMPs), plant-derived antimicrobial substances, probiotics, and bacteriophages. Due to the highest occurrence of research dedicated to those groups of substances authors decided to choose them as currently the most promising methods against foodborne bacterial infections both prevention and treatment. Currently, biological alternatives to antibiotics are increasingly used in prevention and therapies against bacterial pathogenic diseases which are reflected in many scientific publications, clinical trials, and commercialized products described in the article.

### Antimicrobial Proteins

Naturally occurring AMPs have been found in almost every species and are one of the first forms of organism defense against pathogens. AMPs are a very diverse group of molecules, due to that different classification models exist depending on different features such as structure, mechanism of action, or activity. The majority of them have antibacterial activity on which this review will focus. Mechanism of action is a key element in understanding and facilitating further development of AMPs based drugs. There are two major mechanisms: immune modulation and direct killing. Direct killing can be split into membrane killing and non-membrane killing. Membrane killing is also divided into receptor and non-receptor interactions. Bacterial AMPs usually have an affinity to the receptor molecules while most of the eukaryotes AMPs do not need specific receptors to disrupt bacterial membrane. AMPs form pores in a membrane, causing cell death, preceded by leakage of cellular solutes. Their non-receptor mechanism of action also minimizes the risk of developing resistance by bacteria. Bacterial and eukaryotic AMPs share some features, such as small size (15–50 amino acids), positive charge (from + 2 to + 9) with a significant share of cationic arginine or lysine residues, and are hydrophobic (about 50%) or amphiphobic. Their properties facilitate interactions with the negatively charged bacterial cell wall. In the case of Gram-positive bacteria, peptides interact with lipoteichoic acid and peptidoglycan, displacing divalent ions and at the same time providing binding to the negatively charged lipids located at the outside of the cell membrane. In the case of Gram-negative bacteria, AMPs interact with lipopolysaccharides (LPS) and also displace divalent ions. Eukaryotic AMPs have a broader spectrum of antibacterial activity in comparison to bacterial AMPs and they are much less cytotoxic toward eukaryotic cells. They act as the first natural defense; therefore, the highest expression of AMPs is found in the tissues in contact with the environment such as skin, eyes, respiratory epithelium, lungs, intestines, and urinary tract. Moreover, antimicrobial peptides were also isolated from animals, insects, plants, fungi, etc. The diversity of AMPs along with their antibacterial properties could be utilized in the application of these bioactive molecules as promising drug candidates in the pharmaceutical industry. Those peptides can also act against antibiotic-resistant viable but not culturable (VBNC) pathogens, for example, EmPis-1L peptide efficiently eliminates the antibiotic-resistant VBNC state cells of foodborne pathogenic bacteria such as *Escherichia coli* O157 and *Vibrio parahaemolyticus* OS4 [13]. AMPs have also the potential to eradicate bacterial biofilms [14]. Prominent examples of eukaryotic AMPs with therapeutic potential against foodborne pathogens are ll-37 and α-defensin [15], salmine [16], lactoferrin [17], protamine [18], casecidin and isracidin [19], fibrinogen [20], pleurocidin [21], lb-AMP1 peptide [22], α-poly-l-lysine (poly-lys), α-poly-l-arginine (poly-arg) and protamines from herring sperm (clupeine sulfate) and salmon sperm (salmine sulfate) [23].

### Bacteriocins

Bacterial AMPs gained the greatest popularity among AMPs therefore it was needed to describe them as a separate subgroup of potential alternatives. Bacteriocins vary highly in their mode of action and structure; therefore, many different classes and subclasses occur, although in Table 1 we propose one of the simplest and most popular classifications. Lately, it is concluded that bacteriocins should be defined as ribosomally produced multi-functional substances of proteinaceous nature, with pronounced antimicrobial activity at certain concentrations [24]. They are used by bacteria usually to inhibit other closely related species. They have useful features such as the capability of the rapid killing of bacteria and high potency. They often act at picto- or nanomolar concentrations in contrast to other (eukaryotic) AMPs that require higher molar concentrations. Bacteriocins have a narrower spectrum of activity in comparison to antibiotics and eukaryotic AMPs. They usually act only on a few genera or species closely related to the producer. Due to that fact, they have great potential as a new method for fighting with pathogens while keeping alive the probiotic and commensal bacteria. Other advantages of bacteriocins include activity in a wide pH range and tolerance of high thermal stress. Bacteriocins can be considered efficient antimicrobial agents in past used mainly for food preservation. Currently, the evolution of genetic engineering and proteomics gave access to the development of bacteriocin-based alternatives to antibiotics, especially for the resistance to antibiotic pathogenic bacteria strains.

**Table 1:** Simplistic classification of bacteriocins

Type	Features	Mechanism of action
*Origin from Gram-positive Bacteria*		
Class I	Thermostable, polycyclic peptides with a molecular weight of less than 5 kDa, contain in their structure unusual amino acids: lanthionine, 3-methyllanthionine, dehydroalanine	Type A lantibiotics—elongated, flexible molecules whose action is based on the formation of pores in the cytoplasmic membrane of sensitive bacterial cells B-type lantibiotics—rigid, globular molecules with diverse mechanisms of activity. The best known is nisin (*Lactococcus lactis*), which has a bactericidal effect on *Staphylococcus aureus*, *Listeria monocytogenes*, prevents the development of spores, inhibits the growth of vegetative cells *Bacillus*, *Clostridium*
Class II	Non-non-antibiotic bacteriocins—thermostable proteins with a mass less than 10 kDa, divided into 4 subclasses	II A—Pediocin-like bacteriocins—have strong activity against *Listeria ssp.* II B—Two-peptide bacteriocins—to achieve the bactericidal activity, the simultaneous action of both peptides is required II C—Sec-dependent bacteriocins—are secreted using proteins sec II D—Bacteriocins differing in structure, mechanism of action, and secretion from previous
Class III	High molecular bacteriocins—mainly produced by *Lactobacillus* and *Enterococcus* are heat-inactivated	
Class IV	Protein-lipid and protein-carbohydrate complexes require the presence of the lipid or carbohydrate portion in the molecule to achieve full activity	
*Origin from Gram-negative bacteria*		
Colicins	Majority of them found in *E. coli* strains Proteins between 20 and 90 kDa in size	Often consist of a receptor-binding domain, a translocation domain, and a cytotoxic domain Further subclassification can be based on their mechanisms of action either import mechanism (group A and B) or cytotoxic mechanism (nucleases, pore-forming, M-type, L-type)
Microcins	Small, composed of relatively few amino acids	Peptides with a mass less than 5 kDa—post-translational modified, attack intracellular structures Peptides with a molecular weight of 7–10 kDa—not post-translational modified, they work by damaging the cell membrane

Currently, bacteriocins are frequently used as antimicrobial preservatives in food products, in three main forms: bacteria cultures, food products containing bacteriocins in the form of crude fermentate, and finally as partially purified proteins. The bacteria cultures producing a chosen bacteriocin and crude fermentates of bacteriocins are supplemented during food production. Commercial examples of such solutions are BioSafe™ (bacteriocin: Nisin A), Bactoferm™ F-LC (sakacin A and pediocin PA-1/AcH), ALCMix1 (plantaricin and carnocin), Bactoferm™ (Leucocin or Sakacin), MicroGARD® (a mixture of different bacteriocins) [15]. A flagship example of usage of bacteriocin in the inhibition of foodborne pathogens is nisin Z, also in a purified form (commercial product: Nisaplin®, containing 2.5% nisin) [25]. Research showed that orally administered milk with probiotic bacteriocin-producing strains of lactic acid bacteria (LAB), reduced the severity and duration of diarrhea related to *Salmonella* infection in pigs [26]. Bacteriocin pediocin PA-1 reduced vancomycin-resistant Enterococci (VRE) colonization of the intestine of mice [27]. Microcin J25 was shown to reduce infection caused by *Salmonella* in a mouse model [28]. Importantly, bacteriocins can effectively fight biofilm formation [29], for example, bacteriocin derived from *Lactobacillus brevis* DF01 can inhibit the growth of biofilms of two popular foodborne pathogens *E. coli* and *S. enterica* ser. Typhimurium [30]. Moreover, current publications report the effectiveness of LAB producing bacteriocins against antibiotic-resistant Staphylococci [31]. Synergistic use of bacteriocins together with bacteriophages is an interesting possibility, research showed the cooperation of those two therapies against food pathogens, e.g., *L. monocytogenes* [32]. What is interesting, scientists described also the activity of new genetically engineered bacteriocins—integrated enterocin CRL35 and microcin V against clinically isolated enterohemorrhagic *E. coli* and *L. monocytogenes* [33], these findings suggest a further broad potential for the construction of novel bacteriocin antimicrobials. Previous works have shown that AMPs can be produced in plants thanks to currently available genetic engineering tools, this gives a new opportunity for the efficient production of them [34]. AMPs genes expressed in the edible plants could be also an alternative to the addition of antibiotics or other antimicrobials to the feed. Optimization of such technology would allow the prevention and treatment of bacterial foodborne infections, especially in livestock.

As described above success stories highly increased interest in bacteriocins as safe alternatives for antibiotics. The number of AMPs was positively tested for antimicrobial properties against foodborne pathogens, although a continuation of research is necessary to deploy them on a greater scale for their utilization as alternatives to antibiotics. Moreover, it is crucial to evaluate the possibility of increasing bacterial resistance to AMPs. The development of antimicrobial peptides for clinical and commercial use still has some weaknesses, such as high production costs. Yet novel and efficient methods of peptide and protein production, e.g., expression of recombinants in plant cells are described with promising results. Moreover, current studies on bacteriocins are very optimistic and suggest that bio-engineering methods can lead to even more efficient and dedicated applications of bacteriocins. Proteolytic digestion in the intestinal tract may affect bacteriocin activity, due to this their delivery procedures are one of the most challenging parts of their application. For example, the development of micelle nanocarriers for nisin can broaden the applicability of foodborne pathogens treatment [35] as well as encapsulation of bacteriocin can increase its antibacterial activity against pathogens [36]. Thus, research on bacteriocins and AMPs generally will be crucial for further practical use, production, and their application as alternatives to antibiotics by themselves or in synergistic use with probiotics and bacteriophages.

### Bacteriophages

Bacteriophages are bacterial viruses that act as natural predators of bacteria making them a specifically tailored weapon. Even though already at the beginning of the twentieth century, phages were known for their antibacterial properties, only after the emergence of widespread multi antibiotic-resistant strains, research and clinical trials of bacteriophages were undertaken again on a larger scale. Phage therapy relies on the natural mechanism of bacteria cell lysis at the site of infection. However current biotechnological advances fairly broadened the range of potential bacteriophage therapeutics by the usage of purified lytic proteins or engineered phages [37].

Production of bacteriophages has a long history, the best-known phages are in the area of veterinary medicine, especially applicable to living farm animals [38]. In parallel, many clinical trials of phage therapy are now being assayed in humans [39]. They can be effective alone or increase low antibiotic concentration effectiveness, for example in the treatment of *Staphylococcus aureus* [40]. An interesting example of bacteriophage and bacteriocin cooperation is the successful control of *E. coli* both in vitro and in a mouse model by the receptor-binding domains of colicin A with an *E. coli* phage lysin [41]. Many promising results have been obtained in the last several years in defeating pathogens such as *S. aureus* [42], foodborne *E. coli* [43], and *S. enterica* ser. Typhimurium [44]. Interestingly, research on encapsulation of phages allowed to increase the effectiveness of oral therapy for both animals and humans mainly by decreasing gastrointestinal enzyme digestion [45, 46]. Another promising development is the genetic engineering of phages leading to the elimination of immunodominant epitopes and decreasing unwanted immune response during therapy, moreover, such manipulations can create precise bacteriophage variants against targeted pathogen [47, 48]. Veterinary phage vaccines are another interesting concept with encouraging results; however, this approach focuses rather on preventing bacterial infections than its treatment and is based on animal immune response system therefore it would not be further developed in this review [49]. The food industry is the second important area of phage application in particular the meat and raw animal production [50]. For example, a cocktail of specific bacteriophages effectively decreased the amount of *E. coli* bacteria in previously bacteria-inoculated meat samples [51]. Similarly, a mixture of bacteriophages isolated from chicken feces decreased the presence of *S. enterica* ser. Enteritidis on previously inoculated chicken skin [52] and *S. enterica* ser. Typhimurium on porcine skin [53]. Furthermore, bacteriophages can reduce *S. aureus* presence in milk [54]. Additionally, phages can be used also as an antibacterial treatment for plant diseases [55] and as an additive to food packaging [56].

To summarize bacteriophages have a considerable potential to be used as alternatives to antibiotic treatment. What is more, such therapy alone can be considered organic in regards to farm production [57]. Bacteriophages can act also as biosensors in recognizing dangerous VBNC foodborne pathogens, for example, they can distinguish viable and VBNC from dead *Salmonella* cells [58]. Unfortunately, administrative regulations for bacteriophages' use and knowledge about their possible side effects are still rather limited. It is also important to notice that bacteriophage resistance mechanisms can occur as a result of their usage [59]. However, strategies to combat the resistance problem are developed, for example, usage of bacteriophage cocktails (mixtures of more than one bacteriophage) decreases the effect of possible resistance mechanisms by targeting various kinds of mechanisms [60]. Moreover, reports regarding the spreading of foodborne bacterial resistance due to bacteriophages started to emerge causing questions about the security of their free use [61]. Although many successful examples of commercialization: Agriphage (Omnilytics Ltd.), Listex (Micreos, Ltd.), SalmFresh®, ListShield®, and EcoShield® (Intralytix Ltd.), show a foreseeable future for the phage therapy and its administrative regulations [62], provided by more research being carried out to facilitate public perception and safety profile [63].

### Plant-Derived Antimicrobial Compounds

The natural antibacterial properties of plants have been known for centuries; however, only in the last decades, their power to control foodborne pathogens was scientifically confirmed, moreover, they are recognized as promising against antibiotic-resistant bacteria [64]. Plants are a natural and rich source of promising biologically active agents. During evolution, plants had to develop a variety of sophisticated strategies to survive continuous attacks of microorganisms in their environment. Because plants do not have cell-based immune responses, they had to create other methods of fighting bacteria. There are many functional compounds in plants such as polyphenols, phenols, micronutrients, phytochemicals, and essential oils. Those substances of plant origin are sometimes referred to in the literature as phytobiotics, especially in the case of animal nutrition [65]. These organic substances show a natural antioxidant potential as well as antimicrobial properties. It is estimated that there are over 30,000 active antimicrobial substances identified in plants [66]. The majority of essential oils obtained from popular herbs have an antimicrobial activity which is attributed to phenolic and terpenoid compounds and these lipophilic compounds can accumulate in bacterial membranes causing disturbances. Based on their chemical structure they can be classified into a few major groups including essential oils, alkaloids, phenolics (listed in Table 2 together with their mechanism of action). The detailed mechanism involved is an important link contributing to the plant antimicrobial studies. Lately, there are innumerable scientific publications involving plant secondary metabolites and their antibacterial properties. Although only a small fraction of them include analysis of the action mode of antimicrobial plant metabolites for further assessment. Thus, leading to incomplete information on the mode of action which currently prevents substitution of antibiotics by plant secondary metabolites [67].

**Table 2:** General plant antimicrobials classification and its mechanisms

Antimicrobial group	Mechanism
Essential oils	The mechanism of action on an antibiotic or other microorganisms cells is very complex, it involves, among others, denaturation of membrane proteins, the disintegration of the cell membrane, and cell lysis of the microorganism. They can also cause the inactivation of enzymes involved in membrane and wall synthesis, cellular and cell organelles, interfere with cell membrane permeability and electron flow, inhibit the synthesis of DNA, RNA, uptake of proteins and polysaccharides, participation in metabolic processes, and cell division. Essential oils are passed as substances with high lipophilicity, easily penetrate the wall and cell membrane of microorganisms disrupting the integrity and impairment activities essential for the survival of microbe. It is also believed that one of the possible mechanisms of action of essential oils and plant extracts is inhibiting bacterial cell division
Alkaloids	The mode of action of several alkaloid classes such as isoquinoline and polyamine has been studied extensively fairly recently. It has been reported that isoquinoline such as chelerythrine possesses two mechanisms in inhibiting the growth of bacterial cells; through inhibiting the cellular division and nucleic acid synthesis. Isoquinoline inhibits cellular division by tampering with the FtsZ protein, a protein that is essential for the Z ring formation during cellular division. Besides, the synthesis of nucleic acids is also inhibited as isoquinoline inhibits the action of type I topoisomerases; this prevents the translation of antibiotic-resistant genes, increasing bacterial susceptibility toward antibiotics. Polyamine, on the other hand, compromises the integrity and stability of the cell membrane, increasing the membrane permeability via depolarization, leading to leakage of the cytoplasmic contents and later, cell death
Phenolics	Studies have shown that hydrolyzable tannins such as gallotannin are bioactive through the inhibition of glucosyltransferase which is involved in the formation of biopolymers such as DNA, RNA, and protein. Also, hydrolyzable tannins disrupt the peptidoglycan cell wall and cytoplasmic membrane of a drug-resistant strain of *Helicobacter pylori* and *Candida albicans*, leading to the leakage of cellular content and cell death
Organosulfur	The main mechanism involved in the antimicrobial effect is assumed to be the inhibition of thiol-containing enzymes in microorganisms by the rapid reaction of thiosulfinates with thiol groups. Generally, organosulfur compounds show their antimicrobial activity by altering the permeability of microbial cell walls and replacing intracellular and extracellular materials with each other. For example, allicin causes quick and complete inhibition of RNA biosynthesis and additionally a partial inhibition of DNA and protein synthesis

Chosen plants with activity against foodborne bacteria are shown in Table 3. Especially spices and herbs draw attention while thinking about antibacterial potential.

**Table 3:** Chosen plants and their activity against different foodborne pathogens

Plant	Activity against	The main group of antibacterial compounds	References
Garlic (*Allium sativum*)	*E. coli*, *S. aureus, L. monocytogenes* ATCC 7644*, S. aureus* ATCC 25923, *E. coli* ATCC 25922, and *S. enterica* ser. Enteritidi*s* ATCC 13076	Organosulfur compounds (allicin, diallyl sulfides), Phenolic compounds	[78, 79]
Horseradish (*Armoracia rusticana*)	*L. monocytogenes* ATCC 7644, *S. aureus* ATCC 25923, *E. coli* ATCC 25922, *S. enterica* ser. Enteritidis ATCC 13076	Organosulfur compounds (allyl isothiocyanate)	[80]
Basil (*Ocimum basilicum*)	*S. aureus*, *E. coli*, *L. monocytogenes*, and *S. enterica* ser. Enteritidis	Essential oils	[81]
Lemongrass (*Cymbopogon citratus*)	*S. aureus* ATCC 25923, *E. coli* ATCC 25922, *L. monocytogenes* ATCC 19117, *S. enterica* ser. Enteritidis S64	Essential oils	[82]
Clove (*Eugenia caryophillis*)	*L. monocytogenes*, *S*. *enterica* ser. Typhimurium, *E. coli* O157:H7, *Sh. dysenteria*, *S. aureus*, *E. coli*, and *S*. *enterica* ser. Enteritidis	Essential oils	[83]
Bay leaf (*Laurus nobilis*)	*L. monocytogenes* ATCC 19117, *S. enterica* ser. Enteritidis S64, *E. coli* O157:H7, *L. monocytogenes*, *S*. enterica ser. Typhimurium, and *S. aureus*	Essential oils	[84]
Onion (*Allium cepa*)	*E. coli*, *S. aureus*	Polyphenols, flavonoids, essential oils	[85]
Oregano (*Origanum glandulosum*)	*E. coli*, *Lmonocytogenes*, *S. enterica* ser. Typhimurium, *E. coli* O157:H7, *Sh. dysenterie* and *S. aureus*	Essential oils	[86]
Peppermint (*Mentha piperita*)	*S. enterica* ser. Enteritidis	Essential oils	[87]
Black Pepper (*Piper nigrum*)	*S. enterica* ser. Enteritidis	Flavonoids, essential oils	[88]
Rosemary *(Rosmarinus officinalis)*	*L. monocytogenes*, and *S. aureus*	Essential oils	[89]
Sage (*Salvia officinalis*)	*S. enterica* ser. Enteritidis, *L. monocytogenes*, *S. aureus*	Essential oils	[90]
Spanish Lavender (*Lavandula stoechas L*.)	*E. coli* O157:H7, *L. monocytogenes*, *S. enterica* ser. Typhimurium*;*	Essential oils	[91]
Thyme (*Thymus vulgaris*)	*E. coli*, *S. enterica* ser. Enteritidis, *L. monocytogenes*, *S. enterica* ser. Typhimurium, *E. coli* O157:H7, *Sh. dysenteria*, *Bacillus cereus*, and *S. aureus*	Essential oils	[92]
Ginger (*Zingiber officinale*)	*L. monocytogenes*, *S. enterica* ser. Typhimurium, *E. coli* O157:H7, *Sh. dysenterie*, *E. coli*, *S. aureus*	Phenolic compounds, flavonoids (gingerol, shogaol, and zingerone)	[66]

For example allicin, a compound isolated from garlic (*Allium sativum*), is a broad range growth inhibitor for Gram-negative and Gram-positive bacteria. It affects *Escherichia*, *Salmonella* [68], *Streptococcus*, *Staphylococcus*, *Klebsiella*, *Proteus*, and *H. pylori* [69]*.* All serogroups of *E*. *coli*, but especially enterohemorrhagic *E. coli* (serogroup O157) and enterotoxigenic *E. coli* (serogroup O8), were proven to be sensitive to the garlic extract [70]. Plant derivatives can also act as different antibiofilm factors [71]. For example, extracts of *Cuminum cyminum* [72] and *Capparis spinosa* [73] were proven to act as quorum-sensing inhibitors against Gram-negative bacterial pathogens. *Capparis spinosa* extract inhibited motility and interfered with the production of extracellular polymeric substances and biofilm in *E. coli*, *Proteus mirabilis*, *Serratia marcescens*. A growing body of literature has examined citrus plant extracts as rich in various flavonoids, including apigenin, kaempferol, quercetin, and naringenin which can also inhibit biofilm formation in *E. coli* O157:H7 [74] or repress *Salmonella* pathogenicity [75]. Lately, one of the molecules extracted from the tree *Diospyros dendo*, known as ursolic acid, gained much attention due to the successful inhibition of *E. coli* bacterial biofilms in five different *E. coli* hosts (K-12, JM109, C600, EJ500, and JCB495) [76]. The addition of ursolic acid to destabilize biofilms is a promising approach since it is active in low concentrations, this plant-derived compound can be added as a complementary factor to increase the susceptibility of bacterial cells for other antibacterials especially where bacteria are prone to form difficult to target biofilm structures, e.g., in animal production pipelines. Moreover, ursolic acid is non-toxic to hepatocytes. What is important currently there is no evidence that plant extracts could induce a VBNC state in bacteria [77].

Phytochemicals isolated from Chinese and Indian herbs function as immunostimulants, for example in aquacultures [93]. Studies showed that plant compounds and their derivatives could cooperate with probiotics, both due to their negative impact on pathogens and positive impact on gut microflora [94]. However, plant extracts differ from one another as far as the concentrations needed to affect certain bacteria are concerned. It is important to establish common concentrations of plant extracts that have a minimum inhibitory or a minimum bactericidal effect. This could increase the usage of plant extracts as antimicrobial additives in animal feed. However, there are still a few questions that should be examined such as: will animals consume the feed with plant extracts or if there are any side effects of such a diet. Genetically modified plants seem to be an interesting possibility, recombinant DNA technology allows producing food or animal feed that already contains active substances. Minimal processing and avoiding extensive purification costs could provide inexpensive and widely available products [95].

### Probiotics and Prebiotics

Prebiotics are substances that contribute to the growth of microorganisms that are advantageous for their host. The use of probiotics as agents against foodborne pathogens is becoming increasingly popular. The category of probiotic bacteria is wide; however, main groups can be distinguished such as *Lactobacillus*, *Bifidobacteriaceae, and Streptococcus.* Those microorganisms act both as a prevention and control of pathogenic bacteria, supplied to an organism get involved in physiological and immunological processes. In many cases, a pathogen infection is strictly connected to the host’s gut state. To open the door for normal and balanced intestinal colonization, the administration of mixed probiotics and commensal bacteria is recommended. Such an approach and was proven to facilitate the gut microflora function by filling down possible niches for pathogenic bacteria. Secondly, they stimulate the immune system and support defense responses to pathogenic bacteria. Mechanisms of probiotics action are presented in Table 4. Besides that, the vast majority of probiotics also produce bacteriocins.

**Table 4:** Major mechanisms of probiotics action

Major mechanisms of probiotic action	
Common to many types of probiotics	– Protection against colonization – Production of short-chain fatty acids; effect on intestinal passage – Microbiota stabilization/normalization – Acceleration of enterocyte exchange – Competition with pathogens
Common for individual species	– Production of B group vitamins (B1, B2, B6, B8, B12), PP-niacin, folic acid, stimulate the formation of organic acids and amino acids – Production of lactic acid (decreases absorption of toxic substances into the blood) – Direct antagonism – Stabilization of the intestinal barrier – Bile salt metabolism – Enzymatic activity and carcinogen neutralization
Rare mechanisms (specific to individual strains)	– Immune response modulation – Production of specific bioactive substances; endocrine and neurogenic effects

In humans, evidence shows that probiotics can decrease infections and antibiotic-associated diarrhea [96]. Moreover, some strains of probiotic bacteria showed also antimicrobial activity against foodborne pathogens [97]. However, using this technique alone does not seem to lead to the full elimination of pathogenic bacterial infections. Nevertheless, a probiotic approach can reduce cross-contamination and dissemination of infections [98].

In agricultural production, probiotics are already used as a feed additive in aquacultures [99], in chicken [100], pigs [101], and cattle [102]. Research results on probiotic usage in livestock production, show positive growth response in animals and decreased *E. coli* and *Clostridium* amount, bacteria responsible for diarrheal diseases [103]. Moreover, current studies suggest that especially combination of probiotics and antimicrobial plant extracts is more effective in preventing foodborne infections than when they are applied separately [104]. Probiotics can fight the biofilm formation of pathogenic *E. coli* [105]. However, their effectivity of blocking pathogenic bacterial and fungal biofilms creation is based on a probiotic combination [106]. To properly select functional probiotics there can be distinguished major characteristics for the safety and technological usefulness (Table 5). A growing body of literature has examined probiotics being used in animal feed, usually in combination with other compounds, for instance, calves feed with multi-strain *Lactobacillus* probiotic with a combination of phytobiotics with rosmarinic acid improved their health status (reduced occurrence of diarrhea due to reduced amount of *Cryptosporidium*, and *Giardia duodenalis*), starter intake, growth performance, and metabolic status [107].

**Table 5:** Chosen criteria for the selection of probiotic strains for human and animal use

Criteria	Required properties
Health safety	• Natural origin • Isolated from the digestive tract of healthy individuals/animals • Should show a safe use history • Lack of bile acid cleaving skills • No side effects • Lack of antibiotic resistance genes that are located on unstable elements
Functionality	• Competitiveness to the microflora that inhabits the intestinal ecosystem • Survivability, metabolic activity, and growth at destination • Resistant to bile salts • Resistance to the acidic environment of gastric juice • Competitiveness for closely related species • Antagonist activity to foodborne pathogens such as *H. pylori, Salmonella sp., L. monocytogenes, and C. difficile* • Resistance to bacteriocins and acids produced by the endogenous microflora that inhabits the intestinal ecosystem • Adhesion and the ability to colonize specific places in the body
Technological usefulness	• Easy to produce large amounts of biomass • Viability and stability of desirable probiotic bacteria traits during product preparation and distribution • High bacterial storage survival in finished food products • Guaranteeing the desired sensory properties of finished food products • Genetic stability • Resistance to bacteriophages

## Conclusion

The discovery and development of alternatives to antibiotics are currently critically important. The perfect antimicrobial agents should have a possibly narrow spectrum against pathogens and should not cause significant side effects (e.g., diarrhea, colitis, shortage of commensal bacteria). In Table 6, biological antimicrobial agents described in the article have been compared with traditional antibiotics in regards to their safety and toxicity profile. Combining a few non-antibiotic approaches can bring positive results in pathogen prevention. Choosing the right method is a multifactor decision based on efficacy, economics, safety for human and animal health, availability of the treatment, and finally the probability of inducing bacteria resistance.

**Table 6:** Comparison of antibiotics versus biological alternatives against bacterial foodborne pathogens

Characteristic	Antibiotics	Bacteriocins	Bacteriophages	Plant antimicrobials	Probiotics
Synthesis	Enzymes (secondary metabolite)	Ribosomal (primary metabolite)	Bacterial	Secondary metabolites	Bacterial
Bioengineering	Not amendable	Highly amendable	Amendable	Not amendable	Amendable
Spectrum of activity	Mainly broad	Narrow (confined to closely related species)	Narrow	Broad	Medium
Biocompatibility	Toxic	Only toxic at high concentrations	Non-toxic	Non-toxic	Non-toxic
Working concentrations (MIC)	Higher (usually in the micromolar range)	Lower (Often in the pico-nanomolar range)	Lower	Higher (usually in the micromolar range)	Higher (usually in the micromolar range)
Chemical and thermal stability	Tolerate a narrow range of PH and temperature	Tolerate a wide range of pH and temperature	Tolerate a medium range of pH and temperature	Tolerate a wide range of pH and temperature	Tolerate a narrow range of PH and temperature
Adverse effects	Many	None identified	None identified	Few (at high concentrations)	Few (at high concentrations)
Diversity (i.e., in terms of size, microbial target, mode of action, etc.)	Narrow	Broad	Broad	Broad	Medium
Biodegradable	Persistent	Completely metabolized in the human body	Biodegradable	Completely biodegradable	Completely biodegradable
Antibiofilm properties	Resistance	Strong	Medium	Medium	Strong
Cost	Low	High	Medium	Low	Medium
Purification	Possible, high yield	Complicated, low yield	Complicated, high yield	Easy, high yield	Doesn't apply
Specificity	Specific	Specific	Specific	Non-specific	Medium specific
Selectivity	Selective	Non-selective	Non-selective	Non-selective	Non-selective
Bioavailability	Good	Size-dependent	Class dependent	Class dependent	Good
Oral bioavailability	Good	Poor	Medium	Good	Good
Solubility	Variable (low to high)	Low	Low	Variable	Low
Metabolic stability	Slow-fast biotransformation	Low (Fast biotransformation)	Medium stable	Medium stable	Medium stable
Resistance	High occurrence	Possible occurrence	Possible occurrence	No resistance	No resistance

Developing methods as effective as current antibiotic treatments can bring positive changes in countries where those drugs are still approved and overused in livestock production. The majority of the proposed non-antibiotic methods were designed for both humans and animals. Farm production of animals is the first step of the food industry and it is highly important for the safety of the final product and human health. What is more, antibiotic use in the agricultural environment should be reduced as soon as possible to stop the development of resistance by bacteria and to keep the antibiotic effective at least against human infections. Otherwise, in the foreseeable future, we will have to face a dramatic shortage of effective antimicrobial agents for preventing and treatment of bacterial diseases.

While developing novel, more specific methods for the prevention of diseases, it seems highly important to test the pathogens for possible resistance mechanisms [108]. Another emerging challenge in food antimicrobials is inducing a “viable but nonculturable” (VBNC) state by the popular non-biological food sanitizing methods such as temperature, antibiotics, oxygen availability, and others [109]. Bacteria entering the VBNC state represent a serious risk to human health and can induce possible foodborne illnesses. Traditional culture-based methods of foodborne bacteria detection suggest that the tested sample is bacteria-free when in fact pathogens can be present in the VBNC state. Many biological antimicrobial solutions can act as a supplementary treatment to lower the risk of VBNC state in bacteria or inhibit biofilm formation. While testing any new antimicrobial it is important to remember that beyond the CFU method additional methods should be used to check the presence of VBNC state and biofilm formation of bacteria. Furthermore, proper education of individuals using antimicrobials (such as doctors, farmers, food engineers, etc.) is still needed to slow down the processes of gaining resistance by pathogenic bacteria. Also, national and private scientific funding programs should prioritize projects aiming to find efficient alternative strategies for antibiotics. While many countries start to tighten antibiotic regulations, soon it will be necessary to implement alternative substances on a large scale. Due to that, it is extremely significant, especially for the food and breeding industry, to find new, safe, and efficient non-antibiotic solutions against bacterial pathogens.
